# A Conditional Privacy-Preserving Identity-Authentication Scheme for Federated Learning in the Internet of Vehicles

**DOI:** 10.3390/e26070590

**Published:** 2024-07-10

**Authors:** Shengwei Xu, Runsheng Liu

**Affiliations:** 1 Institute of Information Security, Beijing Electronic Science and Technology Institute, Beijing 100070, China; 2 Department of Cryptography Science and Technology, Beijing Electronic Science and Technology Institute, Beijing 100070, China; besti_paradise@163.com

**Keywords:** federated learning, Internet of Vehicles, authentication, certificateless-based cryptography

## Abstract

With the rapid development of artificial intelligence and Internet of Things (IoT) technologies, automotive companies are integrating federated learning into connected vehicles to provide users with smarter services. Federated learning enables vehicles to collaboratively train a global model without sharing sensitive local data, thereby mitigating privacy risks. However, the dynamic and open nature of the Internet of Vehicles (IoV) makes it vulnerable to potential attacks, where attackers may intercept or tamper with transmitted local model parameters, compromising their integrity and exposing user privacy. Although existing solutions like differential privacy and encryption can address these issues, they may reduce data usability or increase computational complexity. To tackle these challenges, we propose a conditional privacy-preserving identity-authentication scheme, CPPA-SM2, to provide privacy protection for federated learning. Unlike existing methods, CPPA-SM2 allows vehicles to participate in training anonymously, thereby achieving efficient privacy protection. Performance evaluations and experimental results demonstrate that, compared to state-of-the-art schemes, CPPA-SM2 significantly reduces the overhead of signing, verification and communication while achieving more security features.

## 1. Introduction

With the rapid development of intelligent transportation systems and Internet of Things (IoT) technology, the Internet of Vehicles (IoV) has become an essential component of smart cities [1]. IoV enables real-time sharing of traffic information and intelligent coordination of vehicles through communication between vehicles and between vehicles and infrastructure. Additionally, with the advancement of machine learning technology, many automotive companies are leveraging machine learning in the IoV to provide more intelligent and efficient services to users [2]. By collecting a large amount of vehicle data to train models, they offer applications such as autonomous driving and traffic flow prediction [3]. However, traditional centralized model training requires gathering vehicle data to the central server for training. Since this vehicle data often contains a significant amount of personal information, such as driving habits, travel routes, home and work locations, many users are concerned about privacy breaches and are reluctant to send their data to the central server [4]. Moreover, recent data security regulations prohibit automotive companies from collecting user data without authorization. To address these privacy concerns, federated learning (FL) has emerged as a solution [5]. FL is a decentralized machine learning approach where multiple clients (such as smartphones, vehicles or other devices) collaboratively train a shared model under the orchestration of a central server while keeping the data localized [6]. Instead of sending raw data to a central server, each client processes the data locally and only shares the model updates (like gradients or parameters) with the central server. The server then aggregates these updates to form a global model. Currently, FL has been widely applied in various IoV scenarios, such as trajectory prediction, advanced driver-assistance systems and traffic flow prediction and management [7].

Although FL addresses the issue of data silos, researchers have found that without proper protection of the transmitted model parameters, attackers can still infer privacy information about user data [8]. Additionally, during the aggregation of parameters by the central server, there is a risk that the server may attempt to infer original data information from the uploaded model parameters. Moreover, due to the open nature of the IoV, attackers can easily eavesdrop on and manipulate messages transmitted between vehicles, gaining access to the vehicles’ real identities and further tracking their behaviors, posing a threat to user privacy [9].

To address the issue of privacy leakage in federated learning, existing solutions are mainly categorized into differential privacy (DP) [10,11,12] and encryption techniques [13,14,15,16,17,18]. DP protects the privacy of original data by adding random noise to model parameters. Wei et al. [10] proposed a differential privacy-based federated learning framework, which achieves different levels of differential privacy protection by adding artificial noise to client parameters before aggregation. Zhao et al. [11] combined DP with federated learning, proposing four localized differential privacy mechanisms to perturb gradients generated by vehicles, thereby preventing privacy leakage. Zhou et al. [12] achieved high-level privacy protection by adding noise and theoretically proved the convergence of their algorithm. Although DP-based solutions have been extended to all machine learning algorithms in deep learning, the added random noise can degrade model accuracy and extend the model convergence time. Encryption-based solutions can be divided into homomorphic encryption and secure multiparty computation (SMC). Zhou et al. [13] combined differential privacy, blinding and Paillier homomorphic encryption to resist model attacks and achieve secure aggregation of model parameters. Ma et al. [14] proposed a dual-trapdoor homomorphic encryption scheme, ShieldFL, which can defend against model poisoning attacks and protect privacy. They also introduced a secure cosine similarity method for Byzantine-robust aggregation. Hijazi et al. [15] introduce four different fully homomorphic encryption (FHE)-based methods for FL, which securely transmit model parameters in encrypted form, thereby enhancing robust privacy and security protection. Zhang et al. [16] present a lightweight dual-server secure aggregation protocol based on secret sharing, achieving both privacy protection and Byzantine robustness. A typical example is secret sharing. This method reduces computational overhead compared to homomorphic encryption but increases the number of communication rounds and communication overhead, thereby hindering the training efficiency of federated learning. Furthermore, encryption-based solutions prevent the cloud server from directly accessing plaintext local model parameters during aggregation. This hinders integration with Byzantine-robust federated learning defense mechanisms [17,18], as existing Byzantine-robust defense mechanisms focus on computing similarities directly on plaintext model parameters. Therefore, it is necessary to research a privacy-preserving federated learning solution suitable for the IoV that can balance efficiency and practicality. 

To ensure the authenticity and integrity of communication data in the IoV, many identity-authentication protocols have been proposed [19]. Currently, existing identity-authentication protocols in the IoV can be primarily categorized into three types: public key infrastructure-based (PKI-based) [20], identity-based (ID-based) [21,22,23,24] and certificateless-based [25,26,27,28]. PKI-based identity-authentication protocols bind a vehicle’s identity to its public key through digital certificates. Vehicles use their private keys to sign messages, and verifiers use the public keys from the vehicle’s digital certificates to verify the signatures. The main drawback of this method is the significant storage and maintenance overhead associated with managing a large number of digital certificates and certificate revocation lists. Identity-based authentication protocols directly use the vehicle’s identity information as the public key, thereby avoiding the overhead of certificate management and maintenance. Zhao et al. [22] proposed an identity-based federated learning collaborative authentication protocol for shared data, achieving efficient anonymous authentication and key agreement between vehicles and other entities. Zhang et al. [23] proposed an ID-based conditional privacy-preserving identity-authentication scheme that does not require bilinear pairings or hash-to-point operations, enabling efficient vehicle authentication. Kanchan et al. [24] proposed a federated learning algorithm based on group signatures, enhancing the protection of node identities. Although ID-based identity-authentication schemes can achieve efficient vehicle authentication, they have the issue of key escrow. Therefore, certificateless identity-authentication schemes have been proposed as a promising solution. However, this approach has a key escrow problem, as the Trusted Authority (TA) has full control over the vehicle’s private keys and can generate legitimate signatures for any vehicle. To address the key escrow issue, certificateless authentication protocols have been proposed. In these protocols, a vehicle’s private key consists of two parts: one part is a secret value selected by the vehicle itself, and the other part is a partial private key generated by TA. Lin et al. [25] proposed a certificateless authentication and key agreement protocol for IoV based on blockchain. This protocol utilizes the decentralized architecture of blockchain to achieve decentralized trusted third-party services, thus mitigating issues such as single-point failure and the risk of trusted third-party disclosure. It aims to achieve efficient authentication between vehicles. Jiang et al. [26] proposed a certificateless anonymous identity-authentication scheme, which aims to anonymize the relationship between terminal identities and data. However, the use of bilinear pairing operations affects authentication efficiency. Ma et al. [27] extended Jiang’s work by proposing a certificateless identity-authentication scheme that does not require bilinear pairing operations and supports batch verification. However, this scheme lacks dynamic member-management capabilities, and the pseudonyms generated by vehicles cannot be dynamically updated. Currently, most existing certificateless authentication protocols use bilinear pairing operations or do not support batch verification, leading to low authentication efficiency. Additionally, most certificateless authentication protocols are independently designed and are not integrated with existing international standard cryptographic algorithms, making them inconvenient for practical application and widespread adoption. Therefore, it is necessary to study an efficient authentication protocol to establish a secure communication environment for the IoV.

To address the aforementioned challenges, we propose a conditional privacy-preserving authentication scheme called CPPA-SM2, which provides secure authentication and privacy protection for vehicle communication and federated learning in the IoV. Specifically, it is based on the fact that if vehicles send messages and participate in training anonymously, even if attackers or the cloud server obtain the plaintext local model parameters and infer some data information, they cannot associate this information with a specific real vehicle identity, thus achieving privacy protection. Our main contributions are as follows:

- We propose a Conditional Privacy-Preserving Authentication scheme, CPPA-SM2, and integrate it with federated learning. Vehicles participate in federated learning training anonymously, obfuscating the link between local model parameters and the vehicle’s real identity, thus achieving privacy protection. Unlike existing privacy-preserving federated learning schemes, it does not require time-consuming encryption operations or add random noise that affects model performance. It maintains the efficiency of federated learning and has the potential to be integrated with Byzantine-robust defense mechanisms.
- CPPA-SM2 is a certificateless identity-authentication scheme based on Elliptic Curve Cryptography, SM2 and the Chinese Remainder Theorem. It can verify the authenticity and integrity of the local model parameters uploaded by vehicles and supports batch verification. Unlike existing certificateless identity-authentication schemes, it integrates with the standard SM2 digital signature algorithm, facilitating practical application. Dynamic member management is achieved through the Chinese Remainder Theorem. When a malicious vehicle is detected in the system, TA can use the system master secret key to trace its real identity and then revoke it from the federated learning system.
- We conducted a security proof and an informal security analysis of the CPPA-SM2 scheme. Additionally, we evaluated its performance through experiments and compared it with other schemes. The experimental results show that CPPA-SM2 can achieve efficient and secure authentication for vehicles while providing privacy protection for federated learning.

The remainder of this paper is organized as follows. Section 2 presents the notation definitions, mathematical background, system model, threat model, security model and design objectives. Section 3 details the implementation of the CPPA-SM2 scheme. Section 4 provides the correctness and security proof of the CPPA-SM2 scheme along with an informal security analysis. Section 5 evaluates the performance of the CPPA-SM2 scheme and compares it with other schemes. Section 6 concludes the paper.

## 2. Preliminaries

In this section, we mainly introduce the preliminary knowledge, system model, threat model, security model and design goals. The relevant symbols used in this paper are explained in Table 1.

### 2.1. Chinese Remainder Theorem

The Chinese Remainder Theorem (CRT) [23,28] is a theorem of number theory that allows one to solve systems of simultaneous congruences with different moduli. It asserts that if one knows the remainders of the division of an integer by several pairwise coprime integers, then one can determine uniquely the remainder of the division of that integer by the product of these integers, under certain conditions.

Let $sk_{1},sk_{2},\ldots ,sk_{n}$ be pairwise co-prime positive numbers and $l_{1},l_{2},\ldots l_{n}$ be any given n positive integers. Then, CRT asserts that the following simultaneous congruence equation

(1) $$X\equiv l_{1}\mathrm{mod}sk_{1},X\equiv l_{2}\mathrm{mod}sk_{2},\ldots ,X\equiv l_{n}\mathrm{mod}sk_{n}$$

has a unique solution X module θ, where $\theta =sk_{1}sk_{2}\cdots sk_{n}=\prod_{i=1}^{n}sk_{i}$, and the X can be obtained by the following equation:

(2) $$X=\sum_{i=1}^{n}l_{i}a_{i}b_{i}(\mathrm{mod}\theta ),$$

where $a_{i}=\theta /sk_{i}$ and $b_{i}={\left(a_{i}\right)}^{-1}\mathrm{mod}sk_{i}$.

### 2.2. Elliptic Curve Cryptosystem

Consider a finite field $F_{p}$ determined by a prime number p. Let $E(F_{p})$ be a set of elliptic curve points over $F_{p}$ defined by the equation $y^{2}=x^{3}+ax+b\mathrm{mod}p$, where $a,b\in F_{p}$ and $(4a^{3}+27b^{2})\mathrm{mod}p\neq 0$. The elliptic curve $E(F_{p})$ includes both scalar multiplication and point addition operations. G is an additive cyclic group with order q. The Elliptic Curve Discrete Logarithm Problem (ECDLP) is defined as follows: Given two random points P,Q∈G on elliptic curve $E(F_{p})$, where $Q=xP,x\in Z_{q}^{*}$, it has been proven that calculating x from Q is computationally difficult. In other words, it is infeasible to find x in polynomial time with a non-negligible probability [29,30].

### 2.3. SM2 Digital Signature Algorithm

The SM2 digital signature algorithm [31] is a public key cryptographic algorithm based on elliptic curve cryptography, developed by the Chinese State Cryptography Administration. It is part of the Chinese National Standards (GB/T 32918.1-2016) [32] and is widely used for secure communications in China. The SM2 digital signature algorithm consists of three main phases: Key Generation, Signature Generation and Signature Verification.

- Key Generation $\left(params)\to (d_{A},P_{A}\right)$: Assume the signer of the message is user A. TA chooses the elliptic curve parameters param=(p,a,b,q,G), selects a random integer $d_{A}\in [1,n-1]$ as the private key and calculates the public key $P_{A}=d_{A}G$ for user A.
- Signature Generation $(params,m,d_{A})\to \sigma_{A}$: Given a message m. A computes $Z_{A}=H(len_{ID_{A}}||ID_{A}||a||b||G||P_{A})$ and $e_{A}=H(Z_{A}||m)$, where $len_{ID_{A}}$ represents two bytes converted from the bit length of user A’s identity $ID_{A}$, a and b are elements in $F_{p}$ that define an elliptic curve over $E(F_{p})$, G denotes the base point in the elliptic curve group G and $P_{A}$ denotes user A‘s public key. Then, A randomly chooses $k_{A}\in [1,n-1]$, calculates $K_{A}=k_{A}\cdot G=(x_{1},y_{1})$ and $r_{A}=(e_{A}+x_{1})\mathrm{mod}q$. Finally A calculates $s_{A}=(k_{A}-r_{A}\cdot d_{A})/(1+d_{A})\mathrm{mod}q$, where $d_{A}$ denotes user A’s private key. User A’s signature on the message m is $\sigma_{A}=(r_{A},s_{A})$.
- Signature Verification $(params,m,\sigma_{A},P_{A})\to true\;or\;false$: Assume the verifier of the signature $\sigma_{A}$ is user B. Given user A’s signature $\sigma_{A}=(r_{A},s_{A})$ on message m, if $r_{A}\notin [1,n-1]\;or\;s_{A}\notin [1,n-1]\;$, B outputs false and exits. Then B computes $Z_{A}=H(len_{ID_{A}}||ID_{A}||a||b||G||P_{A})$, $e_{A}=H(Z_{A}||m)$ and calculates $t_{A}=(r_{A}+s_{A})\mathrm{mod}q$. If $t_{A}=0$, B outputs false and exits. Finally, B calculates $s_{A}G+t_{A}P_{A}=(x_{{\;\;}_{1}}^{’},y_{{\;\;}_{1}}^{’})=K_{A}^{’}$ and $R=(e_{A}+x_{1}^{’})\mathrm{mod}q$. If $R=r_{A}$, B outputs true; otherwise, it outputs false.

### 2.4. System Model

**Figure 1:**
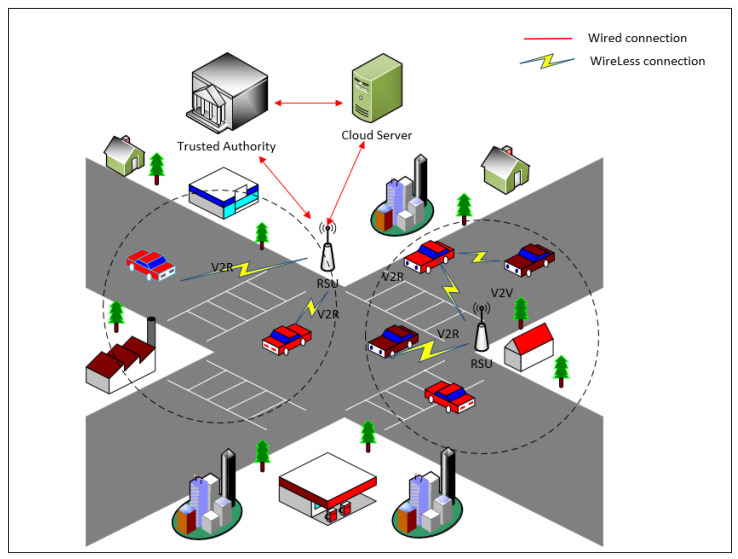
Authentication scheme based on CPPA-SM2 for IoV.

In the IoV, a federated learning system primarily includes four entities: a trusted authority (TA), cloud server (CS), roadside units (RSUs) and vehicles, as shown in Figure 1.

TA: This is a trusted third party, typically the traffic-management department. It is primarily responsible for system initialization, registration of vehicles and RSUs, generating related keys for them and managing identities. In this paper, when a malicious vehicle uploads false local model parameters or forges identity information, the TA can trace its real identity and revoke it from the system.

Vehicles: These are the data owners and participants in federated learning. They use their locally collected data to train the global model received from CS, and then upload the local model parameters. In this paper, vehicles participate in federated learning using pseudonyms, sign the locally trained model parameters and then send them to the nearby RSU.

RSUs: These verify the authenticity and integrity of the local model parameters uploaded by vehicles. They use the FedAvg algorithm [5] to perform local aggregation on these parameters to obtain local aggregation results, which are then uploaded to the cloud server for global aggregation. Additionally, they broadcast the global model issued by TA to the vehicles within their communication range.

CS: Upon receiving the local aggregation results uploaded by RSUs, CS uses FedAvg to perform global aggregation to obtain the global model for the next round of training. The new global model is then distributed to the vehicles to begin the next training round. Through multiple iterations, the performance of the global model can be improved, enabling the cloud server to utilize the results for practical predictions, judgments and applications.

### 2.5. Threat Model and Security Model

In the threat model, CS and RSUs are considered honest-but-curious. This means they will honestly follow the protocol to verify vehicle identities and the authenticity and integrity of model parameters, and they will aggregate local models to obtain the global model [33]. However, they are curious about the private data owned by the vehicles and may attempt to recover the vehicles’ original data and reveal their true identities by analyzing the received model parameters. Therefore, they might pose a threat to vehicle privacy. Vehicles may be malicious and can launch free-riding attacks and data-poisoning attacks by uploading false model parameters. They may also forge identities and signatures to attempt to have fake messages successfully authenticated by RSUs. Additionally, they might try to infer the privacy information of other vehicles. Attackers can fully control the wireless communication channels between vehicles, RSUs, TA and CS. They can intercept messages on the channel, tamper with messages, replay old messages and attempt to impersonate other vehicles to send messages [34].

Based on the aforementioned threats and the certificateless signature security model [27,28,30], our proposed security model is as follows. The hash functions used in this model are assumed to be random oracles.

In the security model, we consider two types of adversaries, $A_{I}$ and $A_{II}$. $A_{I}$ can launch public key-replacement attacks but cannot access system master secret key s. $A_{II}$ can access the system master secret key but cannot perform public key-replacement attacks. Both types of adversaries will engage in two separate games with the challenger C.

Game 1: This security game is executed between $A_{I}$ and C. C initializes the system using the security parameter λ generating system master secret key s and system public parameters param. C secretly keeps s and sends the public parameters to $A_{I}$. $A_{I}$ can perform the following queries.

- Hash queries: Upon receiving a query from $A_{I}$, C returns the corresponding hash values to $A_{I}$.
- Partial-Private-Key-Extract-queries: Upon receiving a query with a pseudonym $PID_{i}$, C returns the partial private key $y_{i}$ of the vehicle to $A_{I}$.
- Public-Key-Extract-queries: Upon receiving a query with a pseudonym $PID_{i}$, C returns the public key $\left(X_{i},Y_{i}\right)$ of the vehicle to $A_{I}$.
- Secret-Value-Extract-queries: Upon receiving a query with a pseudonym $PID_{i}$, C returns the secret value $x_{i}$ of the vehicle to $A_{I}$.
- Public-Key-Replace-queries: Upon receiving a query with $\left(PID_{i},(X_{i}^{’},Y_{i}^{’})\right)$, C replaces public key with the new public key $\left(X_{i}^{’},Y_{i}^{’}\right)$.
- Sign queries: After receiving a query from $A_{I}$ with $\left\{PID_{i,1},PID_{i,2},M_{i},T_{i}\right\}$, C responds with a signature $\sigma_{i}$.
- Forgery: Once $A_{I}$ has completed the desired queries, it outputs $\left\{M_{i}^{*},PID_{i,1}^{*},PID_{i,2}^{*},T_{i}^{*},\sigma_{i}^{*}\right\}$ under the pseudo identity $PID_{{\;\;}_{i}}^{*}$. $A_{I}$ wins the game if the following conditions are met:
- $\sigma_{i}^{*}$ passes verification.
- Partial-Private-Key-Extract-queries oracle has not received the request with $PID_{i}^{*}$.
- Sign queries oracle has not received the request with $\left\{M_{i}^{*},PID_{i,1}^{*},PID_{i,2}^{*},T_{i}^{*}\right\}$.

**Definition** **1.** *CPPA-SM2 is existentially unforgeable under adaptive chosen-identity and chosen-message attacks if no polynomial-time adversary $A_{I}$ can win the above game with non-negligible advantage*.

Game 2: This security game is executed between $A_{II}$ and C. C initializes the system using the security parameter λ generating system master secret key s and system public parameters param. C sends them to $A_{II}$.

- Query: $A_{II}$ can perform all the queries from Game 1 except for Public-Key-Replace-queries.
- Forgery: Once $A_{II}$ has completed the desired queries, it outputs $\left\{M_{i}^{*},PID_{i,1}^{*},PID_{i,2}^{*},T_{i}^{*},\sigma_{i}^{*}\right\}$ under the pseudo identity $PID_{{\;\;}_{i}}^{*}$. $A_{II}$ wins the game if the following conditions are met:
- $\sigma_{i}^{*}$ passes verification.
- Secret-Value-Extract-queries oracle has not received the request with $PID_{i}^{*}$.
- Sign queries oracle has not received the request with $\left\{M_{i}^{*},PID_{i,1}^{*},PID_{i,2}^{*},T_{i}^{*}\right\}$.

**Definition** **2.** *CPPA-SM2 is existentially unforgeable under adaptive chosen-identity and chosen-message attacks if no polynomial-time adversary $A_{II}$ can win the above game with non-negligible advantage*.

### 2.6. Design Goals

Under the security model, CPPA-SM2 primarily has the following design goals:

Anonymity and Privacy-Preserving: CPPA-SM2 should protect the privacy of vehicles participating in federated learning training. No entity other than TA should be able to infer the true identity of the vehicles.

Authenticity and Integrity: CPPA-SM2 should ensure that the local model parameters received by RSUs are from legitimate vehicles and that they have not been tampered with during transmission.

Un-linkability: Attackers cannot link any two messages sent by the same vehicle.

Un-forgeability: Attackers cannot forge signatures of other vehicles on messages, allowing RSUs to successfully verify the signatures.

Non-repudiation: Once a vehicle uploads local model parameters and they are authenticated, the vehicle cannot deny its contribution to the global model.

Forward Security: When a vehicle joins a group, it cannot access communications that occurred before its joining, meaning it cannot participate in previous federated learning training processes of the group.

Backward Security: When a vehicle leaves the group or is revoked by the TA, it cannot participate in the current model training process or access communications that occur after its departure from the group.

In addition to achieving the aforementioned security goals, CPPA-SM2 should also have efficient authentication efficiency and lower communication overhead to adapt to the communication environment of IoV. In particular, when a large number of vehicles participate in federated learning training, RSUs should be able to authenticate them in batches.

## 3. The Proposed Scheme

**Figure 2:**
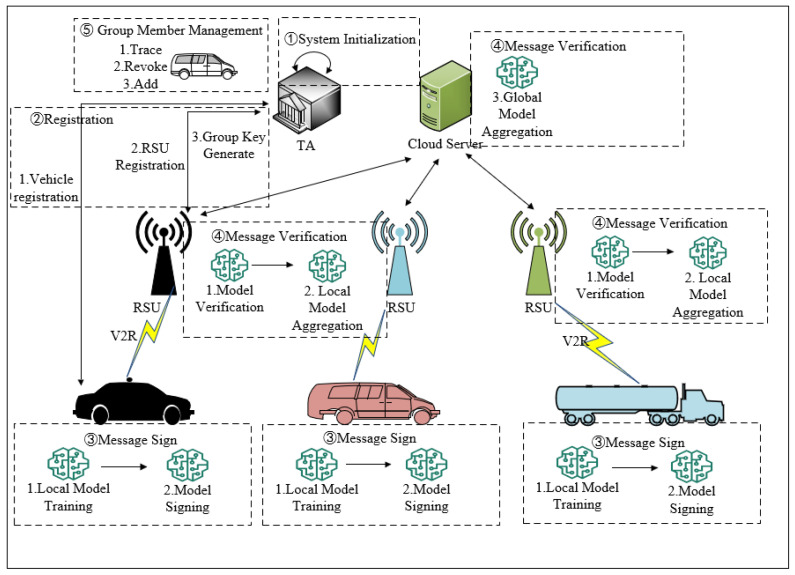
Workflow of CPPA-SM2.

In this section, we present a certificateless conditional privacy-preserving identity-authentication protocol based on CRT and the SM2 digital signature algorithm, named CPPA-SM2. CPPA-SM2 aims to provide privacy protection for vehicles participating in federated learning. It consists of five phases: system initialization, registration, message sign, message verification and group member management. First, TA initializes the system and publishes the system’s public parameters. Then, vehicles and RSUs register with TA before participating in communications. Through registration, they obtain the public and private keys required for subsequent communications. In the message signing phase, vehicles train a model based on their local datasets and then sign the local model parameters before sending them to RSU. RSU, upon receiving the local model parameters from nearby vehicles, verifies the signatures and aggregates the verified local model parameters to obtain a local aggregation result. RSU then sends this local aggregation result to CS for global aggregation, resulting in the next round of the global model. If a malicious vehicle is detected uploading malicious model parameters or forging signatures, TA can trace its identity and revoke it from the system. The overall workflow of CPPA-SM2 is illustrated in Figure 2 and Protocol 1. The details of the scheme are as follows.
**Protocol 1** CPPA-SM2①System InitializationFor TA: 1: Use λ to generate two large prime numbers p and q. 2: Randomly select $s\in Z_{q}^{*}$ and calculates $P_{pub}=s\cdot G$. 3: Choose five one-way hash functions $H_{i}={\left\{0,1\right\}}^{*}\to Z_{q}^{*},i=1,2,3,4,5$. 4: Publish $param=\{p,q,E(F_{p}),G,G,Z_{q}^{*},P_{pub},H_{1},H_{2},H_{3},H_{4},H_{5}\}$. ②RegistrationFor each vehicle: 1: $V_{i}$ randomly selects $x_{i}\in Z_{q}^{*}$, calculates $X_{i}=x_{i}\cdot G$ and send $\left(RID_{i},X_{i}\right)$ to TA. 2: Upon receiving $\left(RID_{i},X_{i}\right)$, TA calculates $h_{i}=H_{1}(X_{i}||P_{pub})$, $y_{i}=s\cdot h_{i}$, $Y_{i}=y_{i}\cdot G$ and randomly selects $sk_{i}\in Z_{q}^{*}$. Then, TA sends $y_{i}$, $Y_{i}$ and $sk_{i}$ to $V_{i}$. 3: $V_{i}$ sets $\left(X_{i},Y_{i}\right)$, $\left(x_{i},y_{i}\right)$ and $sk_{i}$. For each RSU: 1: $RSU_{j}$ sends $ID_{RSUj}$ to TA. 2: TA generates a pair of public and private keys $\left(sk_{RSU_{j}},pk_{RSU_{j}}\right)$ and sends them to $RSU_{j}$. 3. $RSU_{j}$ sets $\left(sk_{RSU_{j}},pk_{RSU_{j}}\right)$. For TA: 1: Calculate $\theta =\prod_{i=1}^{n}sk_{i}$, $a_{i}=\theta /sk_{i}$, $b_{i}={\left(a_{i}\right)}^{-1}\mathrm{mod}sk_{i}$ and set $c_{i}=a_{i}\cdot b_{i}$, $u=\sum_{i=1}^{n}c_{i}$. 2: Randomly pick a group key $K\in Z_{q}^{*}$ and calculate the group public key β=K⋅u and $D_{pub}=K\cdot G$. 3: Sign β, $D_{pub}$ and the K’s valid period $T_{K}$ using its private key $sk_{TA}$ and broadcast the information $\left\{\beta ,D_{pub},SIG_{sk_{TA}}(\beta ||D_{pub}||T_{K})\right\}$ to vehicles and RSUs in $C_{n}$. ③Message SignFor each vehicle: 1: $V_{i}$ trains the global model $W_{global}^{t}$ using its local dataset $D_{i}$ to obtain the local model parameters $W_{i}^{t}$. 2: $V_{i}$ randomly selects $c_{i}\in Z_{q}^{*}$ to generate a pseudo identity $PID_{i}=(PID_{i,1},PID_{i,2})$, where $PID_{i,1}=c_{i}\cdot G$ and $PID_{i,2}=RID_{i}⊕H_{2}(c_{i}\cdot P_{pub})$. 3: $V_{i}$ calculates $Z_{i}=H_{3}(len_{PID_{i,2}}||PID_{i,2}||a||b||G||X_{i})$, $\varphi_{i}=H_{4}(PID_{i,1}||T_{i})$ and $sgk_{i}=y_{i}+Z_{i}\cdot K+x_{i}\cdot \varphi_{i}$. 4: $V_{i}$ randomly selects $k_{i}\in Z_{q}^{*}$, calculates $K_{i}=k_{i}\cdot G=(x_{1},y_{1})$ $e_{i}=H_{5}(Z_{i}||W_{i}^{t}|T_{i})$, $r_{i}=e_{i}+x_{1}\mathrm{mod}q$ and $s_{i}={\left(1+sgk_{i}\right)}^{-1}\cdot (k_{i}-r_{i}\cdot sgk_{i})\mathrm{mod}q$. 5. $V_{i}$ obtains the signature $\sigma_{{\;\;}_{i}}^{t}=(r_{i},s_{i})$ of $W_{i}^{t}$ and sends messages $\left\{W_{{\;\;}_{i}}^{t},\sigma_{{\;\;}_{i}}^{t},(X_{i},Y_{i}),PID_{i},T_{i}\right\}$ to the nearby $RSU_{j}$. ④Message VerificationFor each RSU: 1: Upon receiving the messages $\left\{W_{{\;\;}_{i}}^{t},\sigma_{{\;\;}_{i}}^{t},(X_{i},Y_{i}),PID_{i},T_{i}\right\}$ from $V_{i}$, $RSU_{j}$ first checks the validity of timestamp. If $\Delta T\geq T_{a}-T_{i}$, where $T_{a}$ represents the arrival time, continues; otherwise, discards. 2: $RSU_{j}$ calculates $Z_{i}=H_{3}(len_{PID_{i,2}}||PID_{i,2}||a||b||G||X_{i})$, $e_{i}=H_{5}(Z_{i}||W_{i}^{t}||T_{i})$, $\varphi_{i}=H_{4}(PID_{i,1}||T_{i})$, $t_{i}=r_{i}+s_{i}\mathrm{mod}q$ and $K_{{\;\;}_{i}}^{’}=(x_{1}^{’},y_{1}^{’})=s_{i}\cdot G+t_{i}\cdot [Y_{i}+Z_{i}\cdot D_{pub}+\varphi_{i}\cdot X_{i}]$. 3: $RSU_{j}$ checks the equality of $R=e_{i}+x_{1}^{’}=r_{i}$ for authentication and validity. 4: $RSU_{j}$ uses the FedAvg algorithm to locally aggregate the verified local model parameters $\left\{W_{{\;\;}_{1}}^{t},W_{{\;\;}_{2}}^{t},\ldots ,W_{{\;\;}_{n}}^{t}\right\}$, producing a local aggregation result $W_{RSU_{j}}^{t}\leftarrow FedAvg(W_{i}^{t},n)$. 5: $RSU_{j}$ signs this result with its private key and sends messages $\left\{W_{RSU_{j}}^{t},SIG_{sk_{RSU_{j}}}(W_{RSU_{j}}^{t})\right\}$ to CS. For CS: 1: CS performs a global aggregation on the verified local aggregation results $\left\{W_{RSU_{1}}^{t},W_{RSU_{2}}^{t},\ldots ,W_{RSU_{m}}^{t}\right\}$ to obtain the global model $W_{global}^{t+1}\leftarrow FedAvg(W_{RSU_{j}}^{t},m)$. 2: CS signs the global model with its private key and sends messages $\left\{W_{global}^{t+1},SIG_{sk_{CS}}(W_{global}^{t+1})\right\}$ to the vehicles within the communication group via RSUs. ⑤Group Member ManagementTrace: 1: TA uses the system’s master private key s to recover the vehicle’s true identity $RID_{i}=PID_{i,2}⊕H_{2}(s\cdot PID_{i,1})$. Revoke: 1. TA first removes $c_{i}$ related to $V_{i}$ from u by computing $u^{’}=u-c_{i}$. 2: TA randomly selects a new group key $K^{’}\in Z_{q}^{*}$, calculates new group public keys $\beta^{’}=K^{’}\cdot u^{’}$ and $D_{pub}^{’}=K^{’}\cdot G$, and broadcasts the updated information $\left\{\beta^{’},D_{pub}^{’},SIG_{sk_{TA}}(\beta^{’}||D_{pub}^{’}||T_{K^{’}})\right\}$ to vehicles and RSUs in $C_{n}$. Add: 1. TA randomly selects a new group key $K^{’}\in Z_{q}^{*}$ and calculates $\theta^{’}=\theta \cdot f_{i}$, $a_{i}^{’}=\theta^{’}/f_{i}$, $b_{i}^{’}={\left(a_{i}^{’}\right)}^{-1}\mathrm{mod}sk_{i}$, $c_{i}^{’}=a_{{\;\;}_{i}}^{’}\cdot b_{{\;\;}_{i}}^{’}$ and $u^{’}=\sum_{i=1}^{n}c_{i}^{’}$. 2. TA computes new group public keys $\beta^{’}=K^{’}\cdot u^{’}$ and $D_{pub}^{’}=K^{’}\cdot G$, and broadcasts the updated information $\left\{\beta^{’},D_{pub}^{’},SIG_{sk_{TA}}(\beta^{’}||D_{pub}^{’}||T_{K^{’}})\right\}$ in $C_{n}$. 

### 3.1. System Initialization

TA uses a security parameter λ to generate two large prime numbers p and q, where p>q, $q\leq \left\lceil p/4\right\rceil$. Let $E(F_{p})$ denote an elliptic curve over the finite field $F_{p}$ and G denote a base point on the elliptic curve $E(F_{p})$ with order q. Let G be an additive cyclic group generated by G. TA randomly selects $s\in Z_{q}^{*}$ as the system master secret key and calculates the system public key $P_{pub}=s\cdot G$. Then, TA chooses five one-way hash functions $H_{i}={\left\{0,1\right\}}^{*}\to Z_{q}^{*},i=1,2,3,4,5$. TA secretly holds s and publishes the system’s public parameters $param=\{p,q,E(F_{p}),G,G,Z_{q}^{*},P_{pub},H_{1},H_{2},H_{3},H_{4},H_{5}\}$.

### 3.2. Registration

In the registration phase, both vehicles and RSUs need to register with TA to obtain the relevant keys for subsequent communications. We assume that TA is fully trusted and that the entire registration phase is conducted over a secure channel, eliminating the risk of privacy leaks and security attacks.

#### 3.2.1. Vehicle Registration

For a vehicle $V_{i}$ with its real identity $RID_{i}$, it first randomly selects $x_{i}\in Z_{q}^{*}$ as its secret value and calculates $X_{i}=x_{i}\cdot G$ as its first part of the public key. Then, $V_{i}$ sends $\left(RID_{i},X_{i}\right)$ to TA. Upon receiving $\left(RID_{i},X_{i}\right)$, TA calculates $h_{i}=H_{1}(X_{i}||P_{pub})$, $y_{i}=s\cdot h_{i}$ and $Y_{i}=y_{i}\cdot G$, where $y_{i}$ and $Y_{i}$ serve as $V_{i}$’s partial private key and the second part of the public key. In addition, TA randomly selects a prime number $sk_{i}\in Z_{q}^{*}$ as a secret key for $V_{i}$. Completing these computations, TA returns $y_{i}$, $Y_{i}$ and $sk_{i}$ to $V_{i}$. Upon receiving $y_{i}$, $Y_{i}$ and $sk_{i}$, $V_{i}$ sets $\left(x_{i},y_{i}\right)$ as its full private key, $\left(X_{i},Y_{i}\right)$ as its full public key and uses $sk_{i}$ for subsequent group communications.

#### 3.2.2. RSU Registration

For a roadside unit $RSU_{j}$ with its identity $ID_{RSUj}$, TA generates a pair of public and private keys $\left(sk_{RSU_{j}},pk_{RSU_{j}}\right)$. Then, TA distributes them to $RSU_{j}$. Here, we assume that all vehicles know the public keys of TA and RSUs.

#### 3.2.3. Group Key Generate

To ensure that the uploaded local model parameters come from legitimate vehicles and to support efficient group communication, TA constructs a communication group $C_{n}$ for them based on the secret keys $sk_{i}$ of n vehicles and CRT. TA first calculates $\theta =\prod_{i=1}^{n}sk_{i}$, $a_{i}=\theta /sk_{i}$ and $b_{i}={\left(a_{i}\right)}^{-1}\mathrm{mod}sk_{i}$. TA sets $c_{i}=a_{i}\cdot b_{i}$, $u=\sum_{i=1}^{n}c_{i}$, where i=1,2,…,n. Then, TA randomly picks a group key $K\in Z_{q}^{*}$ and calculates the group public key β=K⋅u and $D_{pub}=K\cdot G$. TA signs β, $D_{pub}$ and the K’s valid period $T_{K}$ using its private key $sk_{TA}$ and broadcasts the information $\left\{\beta ,D_{pub},SIG_{sk_{TA}}(\beta ||D_{pub}||T_{K})\right\}$ to vehicles and RSUs in $C_{n}$. Once receiving the broadcast information, any authorized vehicle in $C_{n}$ can obtain K by performing a modulus operation $K\equiv \beta \mathrm{mod}sk_{i}$ according to CRT.

### 3.3. Message Sign

In the t−th round of training, the vehicle $V_{i}$ trains the global model $W_{global}^{t}$ using its local dataset $D_{i}$ to obtain the local model parameters $W_{i}^{t}$, i.e., $W_{i}^{t}\leftarrow W_{global}^{t}-\eta \nabla L(W_{global}^{t},D_{i})$. Before sending the local model parameter $W_{i}^{t}$ to the nearby $RSU_{j}$, the vehicle $V_{i}$ signs it as follows to ensure the authenticity and integrity of $W_{i}^{t}$.

$V_{i}$ randomly selects $c_{i}\in Z_{q}^{*}$ to generate a pseudo identity $PID_{i}=(PID_{i,1},PID_{i,2})$, where $PID_{i,1}=c_{i}\cdot G$ and $PID_{i,2}=RID_{i}⊕H_{2}(c_{i}\cdot P_{pub})$. Then, $V_{i}$ calculates $Z_{i}=H_{3}(len_{PID_{i,2}}||PID_{i,2}||a||b||G||X_{i})$, $\varphi_{i}=H_{4}(PID_{i,1}||T_{i})$ and signature key $sgk_{i}=y_{i}+Z_{i}\cdot K+x_{i}\cdot \varphi_{i}$, where $len_{PID_{i,2}}$ represents two bytes converted from the bit length of $PID_{i,2}$, a and b are elements in $F_{p}$ that define an elliptic curve over $E(F_{p})$ and $T_{i}$ represents the current timestamp. Next, $V_{i}$ randomly selects $k_{i}\in Z_{q}^{*}$ and calculates $K_{i}=k_{i}\cdot G=(x_{1},y_{1})$ $e_{i}=H_{5}(Z_{i}||W_{i}^{t}|T_{i})$, $r_{i}=e_{i}+x_{1}\mathrm{mod}q$ and $s_{i}={\left(1+sgk_{i}\right)}^{-1}\cdot (k_{i}-r_{i}\cdot sgk_{i})\mathrm{mod}q$. For simplicity, we omit the notation t of $PID_{i}$, $Z_{i}$, $\varphi_{i}$, $sgk_{i}$, $K_{i}$, $e_{i}$, $r_{i}$ and $s_{i}$. Finally, $V_{i}$ obtains the signature $\sigma_{{\;\;}_{i}}^{t}=(r_{i},s_{i})$ of $W_{i}^{t}$ and sends messages $\left\{W_{{\;\;}_{i}}^{t},\sigma_{{\;\;}_{i}}^{t},(X_{i},Y_{i}),PID_{i},T_{i}\right\}$ to the nearby $RSU_{j}$.

### 3.4. Message Verification

#### 3.4.1. Single Message Verification

Upon receiving the messages $\left\{W_{{\;\;}_{i}}^{t},\sigma_{{\;\;}_{i}}^{t},(X_{i},Y_{i}),PID_{i},T_{i}\right\}$ from $V_{i}$, $RSU_{j}$ first checks the validity of the timestamp. If $\Delta T\geq T_{a}-T_{i}$, where $T_{a}$ represents the arrival time, it continues; otherwise, it discards. Then $RSU_{j}$ calculates $Z_{i}=H_{3}(len_{PID_{i,2}}||PID_{i,2}||a||b||G||X_{i})$, $e_{i}=H_{5}(Z_{i}||W_{i}^{t}||T_{i})$, $\varphi_{i}=H_{4}(PID_{i,1}||T_{i})$, $t_{i}=r_{i}+s_{i}\mathrm{mod}q$ and $K_{{\;\;}_{i}}^{’}=(x_{1}^{’},y_{1}^{’})=s_{i}\cdot G+t_{i}\cdot [Y_{i}+Z_{i}\cdot D_{pub}+\varphi_{i}\cdot X_{i}]$. Finally, $RSU_{j}$ checks the equality of $R=e_{i}+x_{1}^{’}=r_{i}$ for authentication and validity.

#### 3.4.2. Batch Messages Verification

When receiving a batch of messages $\left\{W_{1}^{t},\sigma_{1}^{t},(X_{1},Y_{1}),PID_{1},T_{1}\right\}$, $\left\{W_{2}^{t},\sigma_{2}^{t},(X_{2},Y_{2}),PID_{2},T_{2}\right\}$, …, $\left\{W_{n}^{t},\sigma_{n}^{t},(X_{n},Y_{n}),PID_{n},T_{n}\right\}$ from the vehicles $\left\{V_{1},V_{2},\ldots ,V_{n}\right\}$, $RSU_{j}$ first checks the validity of timestamp $T_{i}$, where i=1,2,…,n. If $T_{i}$ is valid, it continues; otherwise, it discards. To prevent confusion attacks while ensuring non-repudiation, CPPA-SM2 uses a set of small exponents $\left\{v_{1},v_{2},\ldots ,v_{n}\right\}$ for batch verification [23,35], where $v_{i}\in [1,2^{t}]$ and t is a small integer. Next, $RSU_{j}$ calculates

(3) $$(x_{1}^{’},y_{1}^{’})=\sum_{i=1}^{n}\left(v_{i}\cdot s_{i}\right)\cdot G+\sum_{i=1}^{n}\left(v_{i}\cdot t_{i}\cdot Y_{i}\right)+\sum_{i=1}^{n}\left(v_{i}\cdot t_{i}\cdot Z_{i}\right)\cdot D_{pub}+\sum_{i=1}^{n}\left(v_{i}\cdot t_{i}\cdot \varphi_{i}\cdot X_{i}\right),$$

and checks whether $R=\sum_{i=1}^{n}\left(v_{i}\cdot e_{i}\right)+x_{1}^{’}=\sum_{i=1}^{n}\left(v_{i}\cdot r_{i}\right)$ holds or not. If true, all messages are valid; otherwise, some of these messages are invalid. The detection algorithm for invalid message signatures has been proposed in [36]. The details of this algorithm are beyond the scope of this paper.

#### 3.4.3. Local Model Aggregation

$RSU_{j}$ uses the FedAvg algorithm to locally aggregate the verified local model parameters $\left\{W_{{\;\;}_{1}}^{t},W_{{\;\;}_{2}}^{t},\ldots ,W_{{\;\;}_{n}}^{t}\right\}$, producing a local aggregation result $W_{RSU_{j}}^{t}\leftarrow FedAvg(W_{i}^{t},n)$, where i∈[1,n] and n denotes the number of vehicles participating in the training within the $RSU_{j}$’s range. It then signs this result with its private key and sends messages $\left\{W_{RSU_{j}}^{t},SIG_{sk_{RSU_{j}}}(W_{RSU_{j}}^{t})\right\}$ to CS. Upon receiving the local aggregation result $W_{RSU_{j}}^{t}$ from RSUs, CS verifies its validity. It then performs a global aggregation on the verified local aggregation results $\left\{W_{RSU_{1}}^{t},W_{RSU_{2}}^{t},\ldots ,W_{RSU_{m}}^{t}\right\}$ to obtain the global model $W_{global}^{t+1}\leftarrow FedAvg(W_{RSU_{j}}^{t},m)$, where j∈[1,m] and m denotes the number of RSUs. CS signs the global model with its private key and sends messages $\left\{W_{global}^{t+1},SIG_{sk_{TA}}(W_{global}^{t+1})\right\}$ to the vehicles within the communication group via RSUs.

### 3.5. Group Member Management

#### 3.5.1. Trace

When $RSU_{j}$ detects that a vehicle $V_{i}$ has uploaded malicious local model parameters or has engaged in identity forgery, it sends the vehicle’s pseudonym $PID_{i}$ to TA. TA then uses the system’s master private key s to recover the vehicle’s true identity $RID_{i}=PID_{i,2}⊕H_{2}(s\cdot PID_{i,1})$.

#### 3.5.2. Revoke

Upon obtaining the true identity $RID_{i}$ of the malicious vehicle $V_{i}$, TA can completely remove it from the federated learning system by revoking its legitimate information from the group. TA first removes $c_{i}$ related to $V_{i}$ from u by computing $u^{’}=u-c_{i}$. Then, TA randomly selects a new group key $K^{’}\in Z_{q}^{*}$, calculates new group public keys $\beta^{’}=K^{’}\cdot u^{’}$ and $D_{pub}^{’}=K^{’}\cdot G$ and broadcasts the updated information $\left\{\beta^{’},D_{pub}^{’},SIG_{sk_{TA}}(\beta^{’}||D_{pub}^{’}||T_{K^{’}})\right\}$ to vehicles and RSUs in $C_{n}$. Upon receiving $\left\{\beta^{’},D_{pub}^{’},SIG_{sk_{TA}}(\beta^{’}||D_{pub}^{’}||T_{K^{’}})\right\}$, the remaining vehicles in $C_{n}$ can use their secret key $sk_{j}$ to compute the updated group key $K^{’}=\beta^{’}\mathrm{mod}sk_{j}$. Since $u^{’}$ no longer contains the legitimate information of $V_{i}$, it cannot compute the new group key $K^{’}$. When a vehicle leaves the communication group $C_{n}$, TA can also revoke it in this way.

#### 3.5.3. Add

When a vehicle $V_{i}$ applies to join the federated learning system, TA randomly selects a new group key $K^{’}\in Z_{q}^{*}$ and calculates $\theta^{’}=\theta \cdot f_{i}$, $a_{i}^{’}=\theta^{’}/f_{i}$, $b_{i}^{’}={\left(a_{i}^{’}\right)}^{-1}\mathrm{mod}sk_{i}$, $c_{i}^{’}=a_{{\;\;}_{i}}^{’}\cdot b_{{\;\;}_{i}}^{’}$ and $u^{’}=\sum_{i=1}^{n}c_{i}^{’}$. Then, TA computes new group public keys $\beta^{’}=K^{’}\cdot u^{’}$ and $D_{pub}^{’}=K^{’}\cdot G$, and broadcasts the updated information $\left\{\beta^{’},D_{pub}^{’},SIG_{sk_{TA}}(\beta^{’}||D_{pub}^{’}||T_{K^{’}})\right\}$ in $C_{n}$. Upon receiving $\left\{\beta^{’},D_{pub}^{’},SIG_{sk_{TA}}(\beta^{’}||D_{pub}^{’}||T_{K^{’}})\right\}$, vehicles in $C_{n}$, it calculates the updated group key $K^{’}=\beta^{’}\mathrm{mod}sk_{i}$.

## 4. Correctness and Security Proof and Analysis

In this section, we first provide a proof of correctness for the proposed scheme. Then, under the random oracle model, we prove the security of the scheme. Finally, we conduct an informal security analysis of the scheme.

### 4.1. Correctness Proof

The correctness verification of the single message signature is ensured by Equations (4) and (5).

(4) $$\begin{array}{ll} K_{{\;\;}_{i}}^{’}=(x_{1}^{’},y_{1}^{’}) & =s_{i}\cdot G+t_{i}\cdot [Y_{i}+Z_{i}\cdot D_{pub}+\varphi_{i}\cdot X_{i}]\\ & =s_{i}\cdot G+(r_{i}+s_{i})[Y_{i}+Z_{i}\cdot D_{pub}+\varphi_{i}\cdot X_{i}]\\ & =s_{i}\cdot G+r_{i}\cdot [Y_{i}+Z_{i}\cdot D_{pub}+\varphi_{i}\cdot X_{i}]+s_{i}\cdot [Y_{i}+Z_{i}\cdot D_{pub}+\varphi_{i}\cdot X_{i}]\\ & =s_{i}\cdot G(1+y_{i}+Z_{i}\cdot K+\varphi_{i}\cdot x_{i})+r_{i}\cdot G(y_{i}+Z_{i}\cdot K+\varphi_{i}\cdot x_{i})\\ & ={\left(1+sgk_{i}\right)}^{-1}\cdot (k_{i}-r_{i}\cdot sgk_{i})\cdot G\cdot (1+sgk_{i})+r_{i}\cdot G\cdot (sgk_{i})\\ & ={\left(1+sgk_{i}\right)}^{-1}\cdot k_{i}\cdot G\cdot (1+sgk_{i})-{\left(1+sgk_{i}\right)}^{-1}\cdot r_{i}\cdot sgk_{i}\cdot G\cdot (1+sgk_{i})+r_{i}\cdot G\cdot (sgk_{i})\\ & =k_{i}\cdot G-r_{i}\cdot sgk_{i}\cdot G+r_{i}\cdot G\cdot (sgk_{i})\\ & =k_{i}\cdot G\\ & =K_{i}=(x_{1},y_{1}) \end{array}$$

(5) $$R=e_{i}+x_{1}^{’}=r_{i}=e_{i}+x_{1}$$

The correctness verification of the batch message signatures is ensured by Equations (6) and (7).

(6) $$\begin{array}{l} \left(\sum_{i=1}^{n}v_{i}\cdot K_{{\;\;}_{i}}^{’}\right)=(x_{1}^{’},y_{1}^{’})=\left(\sum_{i=1}^{n}v_{i}\cdot s_{i}\right)\cdot G+\left(\sum_{i=1}^{n}v_{i}\cdot t_{i}\cdot [Y_{i}+Z_{i}\cdot D_{pub}+\varphi_{i}\cdot X_{i}]\right)\\ \;\;\;\;\;=\left(\sum_{i=1}^{n}v_{i}\cdot s_{i}\right)\cdot G+\left(\sum_{i=1}^{n}v_{i}\cdot (r_{i}+s_{i})\cdot [Y_{i}+Z_{i}\cdot D_{pub}+\varphi_{i}\cdot X_{i}]\right)\\ \;\;\;\;\;=\left(\sum_{i=1}^{n}v_{i}\cdot s_{i}\right)\cdot G+\left(\sum_{i=1}^{n}v_{i}\cdot r_{i}\cdot [Y_{i}+Z_{i}\cdot D_{pub}+\varphi_{i}\cdot X_{i}]\right)+\left(\sum_{i=1}^{n}v_{i}\cdot s_{i}\cdot [Y_{i}+Z_{i}\cdot D_{pub}+\varphi_{i}\cdot X_{i}]\right)\\ \;\;\;\;\;=\left(\sum_{i=1}^{n}v_{i}\cdot s_{i}\cdot G(1+y_{i}+Z_{i}\cdot K+\varphi_{i}\cdot x_{i})\right)+\left(\sum_{i=1}^{n}v_{i}\cdot r_{i}\cdot G(y_{i}+Z_{i}\cdot K+\varphi_{i}\cdot x_{i})\right)\\ \;\;\;\;\;=\left(\sum_{i=1}^{n}v_{i}\cdot {\left(1+sgk_{i}\right)}^{-1}\cdot (k_{i}-r_{i}\cdot sgk_{i})\cdot G\cdot (1+sgk_{i})\right)+\left(\sum_{i=1}^{n}v_{i}\cdot r_{i}\cdot G\cdot (sgk_{i})\right)\\ \;\;\;\;\;=\left(\sum_{i=1}^{n}v_{i}\cdot k_{i}\cdot G\right)-\left(\sum_{i=1}^{n}v_{i}\cdot r_{i}\cdot sgk_{i}\cdot G\right)+\left(\sum_{i=1}^{n}v_{i}\cdot r_{i}\cdot G\cdot (sgk_{i})\right)\\ \;\;\;\;\;=\left(\sum_{i=1}^{n}v_{i}\cdot k_{i}\cdot G\right)\\ \;\;\;\;\;=\left(\sum_{i=1}^{n}v_{i}\cdot K_{i}\right)=(x_{1},y_{1}) \end{array}$$

(7) $$R=\left(\sum_{i=1}^{n}v_{i}\cdot e_{i}\right)+x_{1}^{’}=\left(\sum_{i=1}^{n}v_{i}\cdot r_{i}\right)=\left(\sum_{i=1}^{n}v_{i}\cdot e_{i}\right)+x_{1}$$

Based on the signing and verification process, if the local model parameter $W_{i}^{t}$ and signature $\sigma_{{\;\;}_{i}}^{t}=(r_{i},s_{i})$ transmitted by the vehicle $V_{i}$ have not been tampered with and the signature $\sigma_{{\;\;}_{i}}^{t}=(r_{i},s_{i})$ is generated using the legitimate vehicle’s private key, then according to (4)–(7), RSU can correctly compute that $K_{i}=k_{i}\cdot G=(x_{1},y_{1})=K_{{\;\;}_{i}}^{’}$, thereby making $R=e_{i}+x_{1}^{’}=r_{i}=e_{i}+x_{1}$.

The correctness of legitimate vehicles in $C_{n}$ obtaining the correct group key K is ensured by Equation (8).

(8) $$\begin{array}{l} \beta (\mathrm{mod}sk_{i})\\ =K\cdot u(\mathrm{mod}sk_{i})\\ =K\cdot (a_{1}\cdot b_{1}+\ldots +a_{n}\cdot b_{n})(\mathrm{mod}sk_{i})\\ =K\cdot a_{i}\cdot b_{i}(\mathrm{mod}sk_{i})\\ =K \end{array}$$

When vehicle $V_{i}$ is revoked from the group $C_{n}$ by TA, since $u^{’}=u-c_{i}=(a_{1}\cdot b_{1}+\ldots +a_{n}\cdot b_{n})-a_{i}\cdot b_{i}$, the revoked vehicle will be unable to obtain the correct group key according to Equation (9).

(9) $$\begin{array}{l} \beta^{’}(\mathrm{mod}sk_{i})\\ =K^{’}\cdot u^{’}(\mathrm{mod}sk_{i})\\ =K^{’}\cdot (a_{1}\cdot b_{1}+\ldots +a_{n}\cdot b_{n}-a_{i}\cdot b_{i})(\mathrm{mod}sk_{i})\\ \neq K^{’} \end{array}$$

### 4.2. Security Proof

The security of CPPA-SM2 relies on the ECDLP. In the random oracle model, if there exist adversaries $A_{I}$ and $A_{II}$ who can win games 1 and 2 with non-negligible probabilities, respectively, then there exists a probabilistic polynomial-time simulator that can solve the ECDLP with non-negligible probability.

**Theorem** **1.** *CPPA-SM2 is existentially unforgeable under adaptive chosen-identity and chosen-message attacks against $A_{I}$ with the assumption that ECDLP is hard to resolve.*

**Proof of Theorem** **1.** Let C be the solver of the ECDLP. Suppose that $A_{I}$ can succeed in forging a valid signature by interacting with C. C utilizes $A_{I}$ to solve the ECDLP. Here, we give an ECDLP instance $\left\{G,G^{’}=g\cdot G\right\}$. C executes the simulation to compute g through interacting with $A_{I}$ as follows.

- Setup: On input $\left\{G,G^{’}\right\}$, C sets $P_{pub}=G^{’}$ and returns $\left\{p,q,E(F_{p}),G,Z_{q}^{*},P_{pub},H_{1},H_{2},H_{3},H_{4},H_{5}\right\}$ to $A_{I}$. $A_{I}$ selects $PID_{i}=(PID_{i,1},PID_{i,2})$ as a target vehicle. In addition, C maintains five lists $L=\{PID_{i,1},PID_{i,2},x_{i},y_{i},X_{i},Y_{i}\}$, $L_{H_{1}}=\{h_{i},X_{i},P_{pub}\}$, $L_{H_{3}}=\{Z_{i},len(PID_{i,2}),PID_{i,2},a,b,G,X_{i}\}$, $L_{H_{4}}=\{\varphi_{i},PID_{i,1},T_{i}\}$, $L_{H_{5}}=\{e_{i},Z_{i},M_{i},T_{i}\}$, which are empty initially.
- Query: $A_{I}$ can adaptively make the following queries:
- $H_{1}$-queries: After receiving the queries from $A_{I}$ with $\left\{X_{i},P_{pub}\right\}$, C checks whether $\left\{X_{i},P_{pub}\right\}$ exists in $L_{H_{1}}$. If it does, C returns $h_{i}$ to $A_{I}$. Otherwise, C selects $h_{i}\in Z_{q}^{*}$ randomly and adds $\left\{h_{i},X_{i},P_{pub}\right\}$ to $L_{H_{1}}$. Then, C returns $h_{i}$ to $A_{I}$.
- $H_{3}$-queries: When receiving the queries with $\left\{len(PID_{i,2}),PID_{i,2},a,b,G,X_{i}\right\}$ from $A_{I}$, C checks whether $\left\{len(PID_{i,2}),PID_{i,2},a,b,G,X_{i}\right\}$ exists in $L_{H_{3}}$. If it does, C returns $Z_{i}$ to $A_{I}$. Otherwise, C selects $Z_{i}\in Z_{q}^{*}$ randomly and adds $\left\{Z_{i},len(PID_{i,2}),PID_{i,2},a,b,G,X_{i}\right\}$ to $L_{H_{3}}$. Then, C returns $Z_{i}$ to $A_{I}$.
- $H_{4}$-queries: Upon receiving the queries from $A_{I}$ with $\left\{PID_{i,1},T_{i}\right\}$, C checks whether $\left\{PID_{i,1},T_{i}\right\}$ exists in $L_{H_{4}}$. If it does, C returns $\varphi_{i}$ to $A_{I}$. Otherwise, C selects $\varphi_{i}\in Z_{q}^{*}$ randomly and adds $\left\{\varphi_{i},PID_{i,1},T_{i}\right\}$ to $L_{H_{4}}$. Then, C returns $\varphi_{i}$ to $A_{I}$.
- $H_{5}$-queries: Upon receiving the queries from $A_{I}$ with $\left\{Z_{i},M_{i},T_{i}\right\}$, C checks whether $\left\{Z_{i},M_{i},T_{i}\right\}$ exists in $L_{H_{5}}$. If it does, C returns $e_{i}$ to $A_{I}$. Otherwise, C selects $e_{i}\in Z_{q}^{*}$ randomly and adds $\left\{e_{i},Z_{i},M_{i},T_{i}\right\}$ to $L_{H_{5}}$. Then, C returns $e_{i}$ to $A_{I}$.
- Partial-Private-Key-Extract-queries: After receiving the queries from $A_{I}$ with $PID_{i}=(PID_{i,1},PID_{i,2})$, C checks whether $\left\{PID_{i,1},PID_{i,2},x_{i},y_{i},X_{i},Y_{i}\right\}$ exists in L. If it does, C returns $y_{i}$ to $A_{I}$. Otherwise, C selects $h_{i}\in Z_{q}^{*}$ randomly, computes $y_{i}=s\cdot h_{i}$, $Y_{i}=y_{i}\cdot G$. Then, C sets $x_{i}=X_{i}=⊥$. After that, C adds $\left\{PID_{i,1},PID_{i,2},x_{i},y_{i},X_{i},Y_{i}\right\}$ into L and returns $y_{i}$ to $A_{I}$.
- Public-Key-Extract-queries: After receiving the queries from $A_{I}$ with $PID_{i}=(PID_{i,1},PID_{i,2})$, C checks whether $\left\{PID_{i,1},PID_{i,2},x_{i},y_{i},X_{i},Y_{i}\right\}$ exists in L. If it does, C returns $\left(X_{i},Y_{i}\right)$ to $A_{I}$. Otherwise, C does the Partial-Private-Key-Extract-queries to obtain $y_{i}$. Then, C selects $x\in Z_{q}^{*}$ randomly and computes $X_{i}=x\cdot G$, $x_{i}=x$, $Y_{i}=y_{i}\cdot G$. After that, C adds $\left\{PID_{i,1},PID_{i,2},x_{i},y_{i},X_{i},Y_{i}\right\}$ into L and returns $\left(X_{i},Y_{i}\right)$ to $A_{I}$.
- Secret-Value-Extract-queries: After receiving the queries from $A_{I}$ with $PID_{i}=(PID_{i,1},PID_{i,2})$, C checks whether $\left\{PID_{i,1},PID_{i,2},x_{i},y_{i},X_{i},Y_{i}\right\}$ exists in L. If it does, C returns $x_{i}$ to $A_{I}$. Otherwise, C does the Public-Key-Extract-queries to obtain $\left(x_{i},X_{i},Y_{i}\right)$. After that, C adds $\left\{PID_{i,1},PID_{i,2},x_{i},y_{i},X_{i},Y_{i}\right\}$ into L and returns $x_{i}$ to $A_{I}$.
- Public-Key-Replace-queries: After receiving the queries from $A_{I}$ with $\left\{PID_{i,1},PID_{i,2},X_{i}^{’},Y_{i}^{’}\right\}$, C checks whether $\left\{PID_{i,1},PID_{i,2},x_{i},y_{i},X_{i},Y_{i}\right\}$ exists in L. If it does, C sets $X_{i}=X_{i}^{’}$, $Y_{i}=Y_{i}^{’}$, $x_{i}=y_{i}=⊥$ and updates $\left\{x_{i},y_{i},X_{i},Y_{i}\right\}$ into L. Otherwise, C sets $X_{i}=X_{i}^{’}$, $Y_{i}=Y_{i}^{’}$, $x_{i}=y_{i}=⊥$ and adds $\left\{PID_{i,1},PID_{i,2},x_{i},y_{i},X_{i},Y_{i}\right\}$ to L.
- Sign queries: After receiving the queries from $A_{I}$ with $\left\{PID_{i,1},PID_{i,2},M_{i},T_{i}\right\}$, C retrieves the lists L, $L_{H_{1}}$, $L_{H_{3}}$, $L_{H_{4}}$, randomly selects $v_{i}\in Z_{q}^{*}$, $w_{i}\in Z_{q}^{*}$, $o_{i}\in Z_{q}^{*}$ and sets $s_{i}=v_{i}$, $t_{i}=w_{i}$, $e_{i}=o_{i}$, $K_{i}=(x_{1},y_{1})=s_{i}\cdot G+t_{i}[Y_{i}+Z_{i}\cdot D_{pub}+\varphi_{i}\cdot X_{i}]$, $r_{i}=e_{i}+x_{1}\mathrm{mod}q$. C returns $\sigma_{i}=(r_{i},s_{i})$ to $A_{I}$ and adds $H_{1}$$\left\{e_{i},Z_{i},M_{i},T_{i}\right\}$ into $L_{H_{5}}$. For the output $\sigma_{i}=(r_{i},s_{i})$ of the signature oracle satisfies $K_{{\;\;}_{i}}^{’}=(x_{{\;\;}_{1}}^{’},y_{{\;\;}_{1}}^{’})=s_{i}\cdot G+t_{i}[Y_{i}+Z_{i}\cdot D_{pub}+\varphi_{i}\cdot X_{i}]$, $R=e_{i}+x_{1}^{’}\mathrm{mod}q=r_{i}$.
- Forgery: After all queries have been completed, $A_{I}$ outputs a forged tuple $\left\{M_{i}^{*},PID_{i,1}^{*},PID_{i,2}^{*},T_{i}^{*},\sigma_{i}^{*(1)}\right\}$. C verifies whether $K_{i}{\;\;}^{*}=(x_{1}^{’},y_{1}^{’})=s_{i}^{*}\cdot G+t_{i}^{*}(Y_{i}+Z_{i}^{*}\cdot D_{pub}+\varphi_{i}^{*}\cdot X_{i})$, $R^{*}=e_{i}^{*}+x_{1}^{{’}^{*}}\mathrm{mod}q=r_{i}^{*}$ holds. If it does not hold, C terminates the simulation. Otherwise, C replays the above process by choosing different $H_{1}$, $H_{3}$ and $H_{4}$ based on forking lemma. $A_{I}$ will output three other distinct valid signatures $\sigma_{i}^{*(2)}$, $\sigma_{i}^{*(3)}$ and $\sigma_{i}^{*(4)}$.

Finally, we can obtain four equations as below.

(10) $$k_{i}=s_{i}^{*(j)}+t_{{\;\;}_{i}}^{*(j)}(g\cdot h_{i}+Z_{{\;\;}_{i}}^{*(j)}\cdot K+\varphi_{{\;\;}_{i}}^{*(j)}\cdot x_{i}),\;\mathrm{where}\;j=1,2,3,4.$$

In the above four equations, $k_{i}$, g, K and $x_{i}$ represent the discrete logarithms of $K_{i}$, $P_{pub}$, $D_{pub}$ and $X_{i}$, respectively, which are not known to C. C can obtain the four unknown values by solving the above four linear independent equations, where g is the solution of ECDLP. □

**Theorem** **2.** *CPPA-SM2 is existentially unforgeable under adaptive chosen-identity and chosen-message attacks against* $A_{II}$ *with the assumption that ECDLP is hard to resolve.*

**Proof of Theorem** **2.** Let C be the solver of the ECDLP. Suppose that $A_{II}$ can succeed in forging a valid signature by interacting with C. C utilizes $A_{II}$ to solve the ECDLP. Here, we give an ECDLP instance $\left\{G,G^{’}=g\cdot G\right\}$. C executes the simulation to compute g through interacting with $A_{II}$ as follows.

- Setup: On input $\left\{G,G^{’}\right\}$, C sets $P_{pub}=s\cdot G$ and returns $\left\{p,q,s,E(F_{p}),G,Z_{q}^{*},P_{pub},H_{1},H_{2},H_{3},H_{4},H_{5}\right\}$ to $A_{II}$. $A_{II}$ selects $PID_{{\;\;}_{i}}^{*}=(PID_{{\;\;}_{i,1}}^{*},PID_{{\;\;}_{i,2}}^{*})$ as a target vehicle. In addition, C maintains five lists $L=\{PID_{i,1},PID_{i,2},x_{i},y_{i},X_{i},Y_{i}\}$, $L_{H_{1}}=\{h_{i},X_{i},P_{pub}\}$, $L_{H_{3}}=\{Z_{i},len(PID_{i,2}),PID_{i,2},a,b,G,X_{i}\}$, $L_{H_{4}}=\{\varphi_{i},PID_{i,1},T_{i}\}$, $L_{H_{5}}=\{e_{i},Z_{i},M_{i},T_{i}\}$, which are empty initially.
- Query: C responds to -$H_{i}$-queries (i=1,3,4,5), Partial-Private-Key-Extract-queries, Secret-Value-Extract-queries and Sign queries as in Theorem 1. C responds to Public-Key-Extract-queries as follows.
- Public-Key-Extract-queries: After receiving the queries from $A_{II}$ with $PID_{i}=(PID_{i,1},PID_{i,2})$, C checks whether $\left\{PID_{i,1},PID_{i,2},x_{i},y_{i},X_{i},Y_{i}\right\}$ exists in L. If it does, C returns $\left(X_{i},Y_{i}\right)$ to $A_{II}$. Otherwise, C does the Partial-Private-Key-Extract-queries to obtain $y_{i}$.
- If $PID_{i}=PID_{i}^{*}$, C sets $X_{i}=G^{’}=g\cdot G$, $Y_{i}=y_{i}\cdot G$, $x_{i}=⊥$. C adds $\left\{PID_{i,1},PID_{i,2},x_{i},y_{i},X_{i},Y_{i}\right\}$ into L and sends $\left(X_{i},Y_{i}\right)$ to $A_{II}$.
- If $PID_{i}\neq PID_{i}^{*}$, C chooses $x\in Z_{q}^{*}$ randomly, computes $X_{i}=x\cdot G$, $x_{i}=x$, $Y_{i}=y_{i}\cdot G$. After that, C adds $\left\{PID_{i,1},PID_{i,2},x_{i},y_{i},X_{i},Y_{i}\right\}$ into L and returns $\left(X_{i},Y_{i}\right)$ to $A_{II}$.
- Forgery: After all queries have been completed, $A_{II}$ outputs a forged tuple $\left\{M_{i}^{*},PID_{i,1}^{*},PID_{i,2}^{*},T_{i}^{*},\sigma_{i}^{*(1)}\right\}$. C verifies whether $K_{i}{\;\;}^{*}=(x_{1}^{’},y_{1}^{’})=s_{i}^{*}\cdot G+t_{i}^{*}(Y_{i}+Z_{i}^{*}\cdot D_{pub}+\varphi_{i}^{*}\cdot X_{i})$, $R^{*}=e_{i}^{*}+x_{1}^{{’}^{*}}\mathrm{mod}q=r_{i}^{*}$ holds. If it does not hold, C terminates the simulation. Otherwise, C replays the above process by choosing different $H_{3}$ and $H_{4}$ based on forking lemma. $A_{II}$ will output two other distinct valid signatures $\sigma_{i}^{*(2)}$ and $\sigma_{i}^{*(3)}$.

Finally, we can obtain three equations as below.

(11) $$k_{i}=s_{i}^{*(j)}+t_{{\;\;}_{i}}^{*(j)}(s\cdot h_{i}+Z_{{\;\;}_{i}}^{*(j)}\cdot K+\varphi_{{\;\;}_{i}}^{*(j)}\cdot x_{i}),\;\mathrm{where}\;j=1,2,3.$$

In the above three equations, $k_{i}$, K and $x_{i}$ represent the discrete logarithms of $K_{i}$, $D_{pub}$ and $X_{i}$, respectively, which are not known to C. C can obtain the three unknown values by solving the above three linear independent equations, where $x_{i}$ is the solution of ECDLP.

However, it is difficult to solve the ECDLP in polynomial time. So, under the random oracle model, CPPA-SM2 is existentially unforgeable under adaptive chosen-identity and chosen-message attacks. □

### 4.3. Informal Security Analysis

Anonymity and Privacy-Preserving: In the CPPA-SM2 scheme, vehicles use pseudonyms $PID_{i}=(PID_{i,1},PID_{i,2})$ to communicate with other entities. To obtain the vehicle’s real identity $RID_{i}$, the adversary must compute $RID_{i}=PID_{i,2}⊕H(c_{i}\cdot P_{pub})=PID_{i,2}⊕H(c_{i}\cdot s\cdot G)$. However, due to the hardness of the Computational Diffie–Hellman (CDH) problem, the adversary is unable to obtain $RID_{i}$, thereby protecting the vehicle’s identity privacy. Additionally, since vehicles participate in federated learning using pseudonyms, and these pseudonyms are updated with each message sent, even if external adversaries or RSUs gain access to the plaintext local model parameters, they cannot link them to specific vehicles. This prevents the inference of any private information, thus providing privacy protection during the federated learning process.

Traceability: When a vehicle with malicious behavior is detected, TA can trace its real identity $RID_{i}=PID_{i,2}⊕H(s\cdot PID_{i,1})$ from its pseudonym $PID_{i}=(PID_{i,1},PID_{i,2})$ using the system’s master private key s.

Message integrity and authentication: According to Theorem 1 and Theorem 2, as long as the ECDLP is hard to solve, the CPPA-SM2 scheme is existentially unforgeable under adaptive chosen-identity and chosen-message attacks against the attackers $A_{I}$ and $A_{II}$.

Non-repudiation: Since only the message signer $V_{i}$ can compute the signature key $sgk_{i}$, an adversary cannot forge valid signatures for a specific vehicle identity. Additionally, the TA can execute the Trace algorithm to obtain the vehicle’s real identity. Therefore, once a vehicle’s message passes the signature verification, it cannot be denied.

Un-linkability: Since the vehicle pseudonym identity $PID_{i}$ is generated during the signing process and the random number used in the signature generation process is non-repetitive, each PID in every signature is unique. As a result, any adversary cannot link any number of signatures sent by the same vehicle.

Forward privacy: When a new vehicle joins the group C, the new group key $K^{’}$ is randomly generated by the TA and is independent of the old group key K. Therefore, the newly joined vehicle cannot access the group’s communications prior to joining.

Backward privacy: When a vehicle is revoked or leaves the group, the TA will remove the legitimate information $c_{i}$ associated with that vehicle from u and compute a new group key $K^{’}$ and group public key $\beta^{’}=K^{’}\cdot u$ and $D_{{\;\;}_{pub}}^{’}=K^{’}\cdot G$. Since the revoked vehicle cannot obtain the updated group key $K^{’}$, it cannot access the communications after leaving the group.

Impersonation attack: If an adversary wants to impersonate vehicle $V_{i}$ to the RSUs nearby or other vehicles $V_{j}$, they must generate a valid message $\left\{M_{i},\sigma_{i},(X_{i},Y_{i}),PID_{i},T_{i}\right\}$ that passes the verification algorithm. However, according to Theorem 1 and Theorem 2, it is evident that no polynomial adversary can forge a valid message.

Modification attack: According to Theorem 1 and Theorem 2, any modification of the message $\left\{M_{i},\sigma_{i},(X_{i},Y_{i}),PID_{i},T_{i}\right\}$ can be detected by the verification algorithm. Therefore, the proposed CPPA-SM2 scheme can withstand the modification attack.

Replay attack: In the proposed CPPA-SM2 scheme, vehicles use the current timestamp $T_{i}$ when generating message signatures. Therefore, message verifiers can resist replay attacks by verifying the freshness of the timestamp $T_{i}$.

Collusion attack: Several vehicles would collaborate to try to compute the new group key $K^{’}$ after they left the group. However, since their legitimate information $c_{i}$ has been removed from u, these leaving vehicles cannot conspire to calculate the new group key $K^{’}$.

## 5. Performance Evaluation

In this section, we will evaluate the performance of the proposed CPPA-SM2 scheme from both security features, computation overhead and communication overhead perspectives, and compare and analyze it with the existing works. For bilinear pairings-based CPPA schemes for IoV, we construct a bilinear pairing $\bar{e}:G_{1}\times G_{1}\to G_{T}$, where $G_{1}$ is an additive group generated by a point $\bar{G}$ with the order $\bar{q}$ on the super singular elliptic curve $\bar{E}:y^{2}=x^{3}+x\mathrm{mod}\bar{p}$ with embedding degree 2, $\bar{p}$ is a 512-bit prime number, $\bar{q}$ is a 160-bit prime number. For ECC-based CPPA schemes for IoV, we construct an additive group G generated by a point G with the order q on a non-singular elliptic curve $E:y^{2}=x^{3}+ax+b\mathrm{mod}p$, where p,q are two 256-bit prime numbers and $a,b\in Z_{p}^{*}$. We calculate the execution time of basic cryptographic operations using the MIRACL library in VS 2019 with Windows 11 operating system over an Intel(R) Core(TM) i7-9750H CPU @ 2.60GHz, as shown in Table 2.

### 5.1. Computation Costs

**Figure 3:**
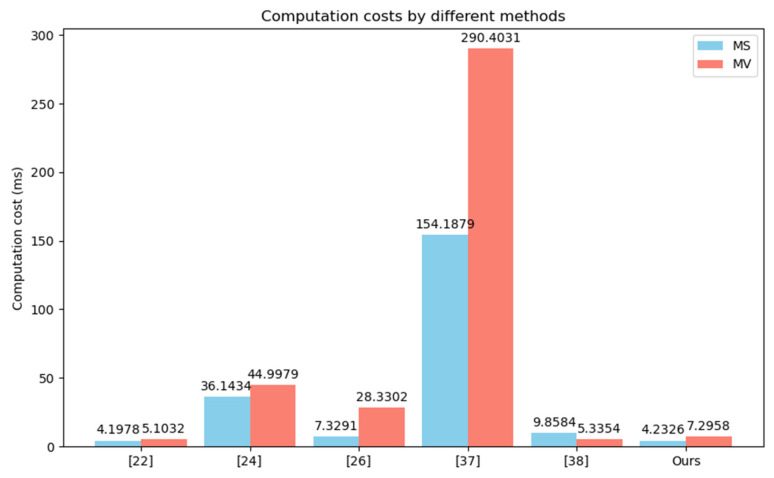
Comparison of computation costs.

**Figure 4:**
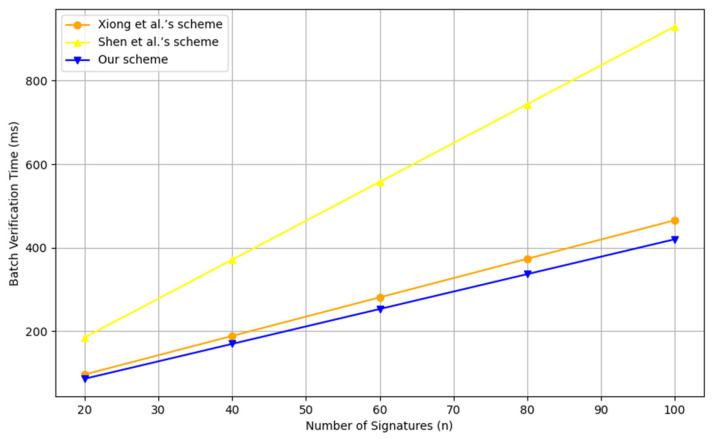
Comparison of the scheme proposed by [28,39], and our scheme in batch validation time.

We compared the computational costs of the CPPA-SM2 scheme with other relevant schemes in terms of signature generation, single signature verification, batch verification and member management, as shown in Table 3 and Table 4, and Figure 3 and Figure 4, where “-” indicates that the property is not considered in the scheme, MS denotes the message sign and MV denotes the message verification.

**Table 3:** Analysis of computation costs for different schemes.

Scheme	MS	MV	Trace	Revoke
[22]	$2T_{⊕}+2T_{em}+4T_{h}$	$2T_{⊕}+2T_{em}+7T_{h}$	-	Revocation list
[24]	$T_{h}+4T_{bp}+4T_{bpe2}+6T_{e}+9T_{m}$	$T_{h}+2T_{m}+4T_{bpe2}+5T_{bp}+8T_{e}$	$T_{bpe1}$	Revocation list
[26]	$2T_{h}+5T_{bpe1}$	$T_{i}+T_{bpe1}+T_{bpe2}+T_{h}+T_{DE}+T_{bpm2}+3T_{bp}$	O(1)	Revocation list
[37]	$T_{⊕}+2T_{h}+3T_{mtp}+4T_{bpm1}+6T_{bpe1}$	$T_{⊕}+T_{bpm1}+2T_{h}+3T_{bpe1}+3T_{bpm2}+5T_{bp}+5T_{mtp}$	$T_{DE}$	-
[38]	$2T_{h}+2T_{ea}+3T_{m}+6T_{em}$	$T_{h}+3T_{em}+4T_{ea}$	$T_{em}+T_{ea}$	Revocation list
Ours	$T_{⊕}+T_{i}+2T_{em}+4T_{m}+4T_{h}$	$3T_{h}+3T_{ea}+4T_{em}$	$T_{h}+T_{⊕}$	$T_{\mathrm{mod}}$

**Table 4:** Comparison of batch-verification costs.

Scheme	Batch Verification Time
[28]	$4nT_{h}+(2n+3)T_{em}+(3n+1)T_{ea}$
[39]	$nT_{bp}+nT_{bpe2}+(3n-2)T_{bpm1}$
Ours	$3nT_{h}+(2n+2)T_{em}+(2n+1)T_{ea}$

Zhao et al. scheme [22] offers relatively low computational overhead, but RSU needs to send a request to TA for each identity verification, and there is a key escrow issue. In Kanchan et al. scheme [24] based on bilinear pairings, group signature is used instead of an individual signature for message authentication, and the group manager achieves tracing of malicious vehicles. Generating a group signature requires performing $T_{h}+4T_{bp}+4T_{bpe2}+6T_{e}+9T_{m}$. Verifying the group signature requires performing $T_{h}+2T_{m}+4T_{bpe2}+5T_{bp}+8T_{e}$, resulting in a relatively high computational overhead. In Jiang et al. scheme [26], similarly, bilinear pairing operations are used, requiring $2T_{h}+5T_{bpe1}$ computations to generate a signature and $T_{i}+T_{bpe1}+T_{bpe2}+T_{h}+T_{DE}+T_{bpm2}+3T_{bp}$ computations to verify the signature. In Yang et al. scheme [37], generating a signature requires performing $T_{⊕}+2T_{h}+3T_{mtp}+4T_{bpm1}+6T_{bpe1}$. To verify the signature, $T_{⊕}+T_{bpm1}+2T_{h}+3T_{bpe1}+3T_{bpm2}+5T_{bp}+5T_{mtp}$ operations are needed. Due to the involvement of bilinear pairings and hash-to-point mappings, this method incurs the highest computational overhead. In Lin et al. scheme [38], a vehicle calculates $2T_{h}+2T_{ea}+3T_{m}+6T_{em}$ to generate the anonymous public keys and a signature. Upon receiving the signature, RSU verifies it by performing $T_{h}+3T_{em}+4T_{ea}$. Additionally, Zhao et al. scheme [22], Kanchan et al. scheme [24], Jiang et al. scheme [26] and Lin et al. scheme all require maintaining a revocation list for revocation purposes, which incurs additional lookup and maintenance overhead. CPPA-SM2 does not require bilinear pairings or hash-to-point mappings, relying only on basic ECC operations, thus reducing computational costs. Specifically, when a vehicle sends a message, it first generates an unlinkable pseudonym $PID_{i}$ by performing one $T_{em}$, one $T_{⊕}$ and one $T_{h}$. Then, it generates the signature by performing three $T_{h}$, one $T_{em}$, four $T_{m}$ and one $T_{i}$. Therefore, the computation cost for signature generation is $T_{⊕}+T_{i}+2T_{em}+4T_{m}+4T_{h}$. To authenticate the message sent by the vehicle, the RSU, upon receiving the message, needs to perform $3T_{h}+3T_{ea}+4T_{em}$. Therefore, the total computation cost for signature generation and signature verification in CPPA-SM2 is $T_{⊕}+T_{i}+3T_{ea}+4T_{m}+6T_{em}+7T_{h}$. When RSU receives messages sent from n vehicles, it performs batch verification of the messages by executing $(2n+1)T_{ea}+(2n+2)T_{em}+3nT_{h}$. To test the effectiveness of batch verification, we conducted experimental comparisons between CPPA-SM2 and Xiong et al. scheme [28] and Shen et al. scheme [39]. In batch verification, the RSU will verify the n messages received simultaneously from n vehicles, meaning n represents both the number of signatures received by the RSU at the same time and the number of vehicles. In the experiment, we tested with n set to 20, 40, 60 and 100, respectively. In CPPA-SM2, when RSU simultaneously receives n messages from n vehicles, it needs to compute three $T_{h}$, two $T_{em}$ and two $T_{ea}$ for each vehicle. Finally, it performs two $T_{em}$ and one $T_{ea}$ to verify multiple messages. Therefore, the total cost of batch verification is $3nT_{h}+(2n+2)T_{em}+(2n+1)T_{ea}$. In Xiong et al. scheme [28], it performs four $T_{h}$, two $T_{em}$ and three $T_{ea}$ for each vehicle. Then, it also executes three $T_{em}$ and one $T_{ea}$. Therefore, the total cost of batch verification is $4nT_{h}+(2n+3)T_{em}+(3n+1)T_{ea}$. In Shen et al. scheme [39], RSU invokes one exponent operation, one bilinear pairing and one multiplication to confirm the equation $m=e(\eta ,pk_{i})e{\left(P,P\right)}^{-r_{2}}$. Its batch verification is based on $\prod_{n}e(\eta_{n},pk_{n})e{\left(P,P\right)}^{-r_{2,n}}=\prod_{n}m_{n}$, which needs n times $T_{bp}$, n times $nT_{bpe2}$ and $(3n-2)T_{bpm1}$. The results are shown in Table 4 and Figure 4. From the experimental results, it can be seen that the batch-verification performance of our scheme is better than these two schemes. In terms of tracing cost, Kanchan et al. scheme [24], Yang et al. scheme [37], Lin et al. scheme [38] and CPPA-SM2 are 1.3451 ms, 0.1759 ms, 1.6320 ms and 0.3027 ms, respectively. All these approaches can achieve fast identity tracing. But in terms of revocation, all schemes except CPPA-SM2 utilize revocation lists, leading to additional maintenance and lookup overheads, while CPPA-SM2 only requires a single modular operation to efficiently revoke vehicles. Therefore, overall, compared to other schemes, CPPA-SM2 not only reduces the computational costs of signature generation and verification, and supports batch verification, but it also achieves efficient tracing and revocation of vehicles while preserving vehicle privacy.

### 5.2. Communication Costs

**Figure 5:**
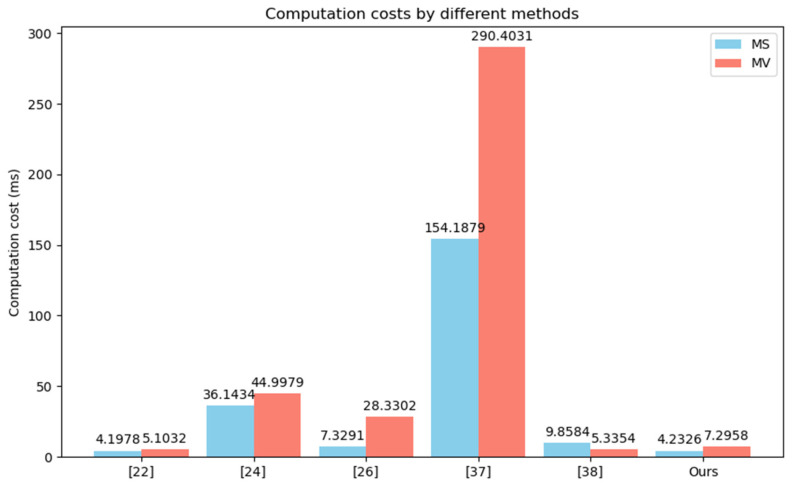
Comparison of communication costs.

We compared the communication costs of CPPA-SM2 with other schemes, mainly including the following: the size of single signature (SSS), the total number of transmitted messages (NTMs), their sizes (STMs) and the number of interactions (NIs). The results are shown in Table 5 and Figure 5. In Zhao et al. scheme [22], to complete the authentication, interaction is required four times, making it the highest number of interactions. Its total computational cost is 476 bytes. The communication overhead for the group signature $\left\{D_{1},D_{2},D_{3},c,s_{\alpha },s_{\beta },s_{x},s_{\delta_{1}},s_{\delta_{2}}\right\}$ generated in Kanchan et al. scheme [24] is the highest, at 576 bytes. Jiang et al. scheme [26], Yang et al. scheme [37] and CPPA-SM2 all require only one interaction to complete message authentication. In Lin et al. scheme [38], vehicles need to transmit $\left\{\sigma_{n},k_{n},U_{n},D_{n},Z_{n}^{’}\right\}$ for message authentication, with a total size of 480 bytes. In CPPA-SM2, the generated signature, denoted as $\sigma_{i}=(r_{i},s_{i})$, consists of two elements from $Z_{q}^{*}$; hence, its size is merely 64 bytes. To authenticate the signature, three additional messages $\left\{PID_{i},(X_{i},Y_{i}),T_{i}\right\}$ of size 228 bytes need to be transmitted, resulting in a total transmission cost of 292 bytes. In Yang et al. scheme [37], The generation of a single signature is denoted as $C_{i}=\{R_{i},c_{i},s_{i}\}$, where $R_{i}$, $c_{i}$ and $s_{i}$ belongs to $G_{1}$; thus, the size of $C_{i}$ is 384 bytes.

In Lin et al. scheme [38], the obtained signature is denoted as $\left\{c_{i},z_{i,1},z_{i,2},R_{i,1},R_{i,2}\right\}$, with a length of 224 bytes. Additionally, to resist replay attacks, $\left\{ts_{i},APK_{a}^{1},APK_{a}^{2}\right\}$ are also sent, making the total message length for transmission 356 bytes. From the experimental results, it can be observed that CPPA-SM2 has the smallest signature size and total cost of transmitting messages. This makes it more suitable for operation in bandwidth-constrained vehicular networking environments.

### 5.3. Security Features

We compared the security features (SFs) satisfied by these schemes, including the following: 1: anonymity; 2: traceability; 3: authenticity; 4: integrity; 5: non-repudiation; 6: un-linkability; 7: forward security; 8: backward security; 9: key escrow-free; 10: batch verification; 11: revocability; 12: dynamic member management; and 13: un-forgeability. The results are shown in Table 6, where 1–13 represent these security features in order, with √ indicating that the security feature is met and × indicating that it is not met. From the results, it can be seen that all schemes achieve 1: anonymity, 3: authenticity, 4: integrity and 6: un-linkability. Zhao et al. scheme [22], Kanchan et al. scheme [24], Jiang et al. scheme [26] and CPPA-SM2 use digital signatures to verify the authenticity and integrity of the local model parameters uploaded by vehicles. However, in Zhao et al. scheme [22] and Kanchan et al. scheme [24], since TA possesses all users’ private keys, there is a key escrow issue. Jiang et al. scheme [26] satisfies most of the security features; however, it uses a revocation list for identity management, resulting in additional verification and maintenance overhead. Furthermore, it does not support 12: dynamic member management. To achieve 6: un-linkability, Yang et al. scheme [37] and Lin et al. scheme [38] use a set of pseudonyms to hide real identities, whereas CPPA-SM2 achieves 6: un-linkability by randomly generating pseudonyms each time a signature is made. Overall, compared to these schemes, CPPA-SM2 achieves more comprehensive security attributes, supports 10: batch verification and 12: dynamic member management, and has lower computational and communication costs.

Overall, compared to the state-of-the-art scheme, Jiang et al. scheme [26], CPPA-SM2 reduces the cost of single signature generation and verification by 42.25% and 74.25%, respectively. In terms of communication overhead, CPPA-SM2 reduces it by 60% and 39.17%, respectively. While the performance of CPPA-SM2 in batch verification is not as good as Jiang et al. scheme [26], it supports dynamic member management, enabling efficient member addition and revocation, which results in increased batch-verification costs.

## 6. Conclusions

In this paper, we propose a conditional privacy-preserving identity-authentication protocol that provides privacy protection for vehicles participating in federated learning in the IoV. Unlike most existing privacy-preserving federated learning schemes, it does not require complex cryptographic operations or the introduction of random noise. Instead, it achieves privacy protection by using dynamic pseudonyms to obscure the connection between model parameters and the real identities of vehicles, thereby maintaining federated learning efficiency.

Moreover, CPPA-SM2 is a certificateless authentication scheme based on ECC, CRT and the SM2 digital signature algorithm. It enables efficient identity authentication and dynamic member management, and supports batch verification. Security proofs and analyses demonstrate that it can ensure the authenticity and integrity of local model parameters, achieving secure vehicle authentication. Experimental results show that, compared to existing advanced schemes, CPPA-SM2 offers high computational efficiency and low communication overhead. Additionally, its integration with standard algorithms endows it with the potential for widespread application.

However, the focus of this paper is on identity-authentication schemes and privacy protection in the federated learning process. There are still some malicious clients in the federated learning process that may launch data-poisoning attacks by uploading malicious local model parameters, thereby affecting the performance of the global model. Therefore, future research could integrate Byzantine robust detection schemes to achieve privacy-preserving Byzantine robust federated learning. Additionally, with the development of post-quantum algorithms, the ECDLP may be efficiently solved by post-quantum algorithms, making ECC-based authentication schemes no longer secure. Future work can explore quantum-resistant identity-authentication schemes, such as lattice-based cryptography.

## Figures and Tables

**Table 1:** Notations and definitions used.

Notations	Definition
λ	Security parameter
s	System master secret key
$P_{pub}$	System public key
$\left(pk_{TA},sk_{TA}\right)$	TA’s public and private key pair
$\left(pk_{RSU},sk_{RSU}\right)$	RSU’s public and private key pair
$V_{i}$	The i-th vehicle
K	Group key
$\left(\beta ,D_{pub}\right)$	Group public key
$\left(X_{i},Y_{i}\right)$	$\mathrm{Vehicle}\;V_{i}$’s full public key
$\left(x_{i},y_{i}\right)$	$\mathrm{Vehicle}\;V_{i}$’s full private key
$sk_{i}$	$\mathrm{Vehicle}\;V_{i}$’s secret key
$RID_{i}$	$\mathrm{Vehicle}\;V_{i}$’s real identity
$PID_{i}=(PID_{i,1},PID_{i,2})$	An pseudo-identity of vehicle $V_{i}$
$T_{i}$	Current timestamp
$T_{a}$	Arrival time
ΔT	The validity period of the pseudo-identity
$T_{K}$	The validity period of the group key
$H_{1},H_{2},H_{3},H_{4},H_{5}$	Five one-way hash functions
$sgk_{i}$	$\mathrm{The}\;\mathrm{signature}\;\mathrm{key}\;\mathrm{for}\;\mathrm{vehicle}\;V_{i}$
||	Concatenation operation
SIG	Signature algorithm
$W_{i}^{t}$	$\mathrm{The}\;\mathrm{local}\;\mathrm{model}\;\mathrm{parameters}\;\mathrm{of}\;\mathrm{vehicle}\;V_{i}$ in round t
$W_{RSU_{j}}^{t}$	$\mathrm{The}\;\mathrm{local}\;\mathrm{model}\;\mathrm{parameters}\;\mathrm{aggregated}\;\mathrm{by}\;RSU_{j}$ in round t
$W_{global}^{t+1}$	The global model for round t+1

**Table 2:** Execution time of basic cryptographic operations and element size.

Symbols	Meanings	Time (ms)/Size (Byte)
$T_{inverse}$	$\mathrm{Time}\;\mathrm{of}\;\mathrm{module}\;\mathrm{inverse}\;\mathrm{on}\;Z_{q}^{*}$	0.0181 ms
$T_{\mathrm{mod}}$	$\mathrm{Time}\;\mathrm{of}\;\mathrm{mod}\;\mathrm{operation}\;\mathrm{on}\;Z_{q}^{*}$	0.0020 ms
$T_{e}$	$\mathrm{Time}\;\mathrm{of}\;\mathrm{module}\;\mathrm{exponential}\;\mathrm{on}\;Z_{q}^{*}$	0.0434 ms
$T_{m}$	$\mathrm{Time}\;\mathrm{of}\;\mathrm{module}\;\mathrm{multiplication}\;\mathrm{on}\;Z_{q}^{*}$	0.0044 ms
$T_{SE}$	Encryption time of AES algorithm	10.0761 ms
$T_{DE}$	Decryption time of AES algorithm	0.1759 ms
$T_{⊕}$	Time of XOR operation	0.0009 ms
$T_{bp}$	Time of bilinear pairing	8.7985 ms
$T_{bpm1}$	$\mathrm{Time}\;\mathrm{of}\;\mathrm{multiplication}\;\mathrm{on}\;\mathrm{bilinear}\;\mathrm{group}\;G_{1}$	0.1361 ms
$T_{bpe1}$	$\mathrm{Time}\;\mathrm{of}\;\mathrm{exponential}\;\mathrm{on}\;\mathrm{bilinear}\;\mathrm{group}\;G_{1}$	1.3451 ms
$T_{bpm2}$	$\mathrm{Time}\;\mathrm{of}\;\mathrm{multiplication}\;\mathrm{on}\;\mathrm{bilinear}\;\mathrm{group}\;G_{2}$	0.0069 ms
$T_{bpe2}$	$\mathrm{Time}\;\mathrm{of}\;\mathrm{exponential}\;\mathrm{on}\;\mathrm{bilinear}\;\mathrm{group}\;G_{2}$	0.0869 ms
$T_{em}$	Time of scalar multiplication on ecliptic curve group G	1.4944 ms
$T_{ea}$	Time of point addition on ecliptic curve group G	0.1376 ms
$T_{h}$	Time of one-way hash function	0.3018 ms
$T_{mtp}$	Time of hash mapped to point	48.3228 ms
|T|	Size of timestamp	4 bytes
|ID|	Size of ID	8 bytes
|AES|	The ciphertext size of AES algorithm	32 bytes
|G|	Size of elements on elliptic curve G	64 bytes
$\left|G_{1}\right|$	Size of elements on bilinear group $G_{1}$	128 bytes
$\left|G_{2}\right|$	Size of elements on bilinear group $G_{2}$	128 bytes
$\left|Z_{q}^{*}\right|$	$\mathrm{Size}\;\mathrm{of}\;\mathrm{elements}\;\mathrm{on}\;Z_{q}^{*}$	32 bytes
|H|	Output size of hash function	32 bytes

**Table 5:** Comparison of communication costs for different schemes.

Scheme	SSS	NTM	STM	NI
[22]	$\left|ID|+|G|+|T|+2|Z_{q}^{*}\right|$	4	$2|ID|+2|G|+3|T|+10|Z_{q}^{*}|$	4
[24]	$\left|G_{2}|+2|G_{1}|+6|Z_{q}^{*}\right|$	9	$\left|G_{2}|+2|G_{1}|+6|Z_{q}^{*}\right|$	2
[26]	$\left|G_{1}|+|Z_{q}^{*}\right|$	5	$3|G_{1}|+3|Z_{q}^{*}|$	1
[37]	$3|G_{1}|$	2	$3|G_{1}|$	1
[38]	$2|G|+3|Z_{q}^{*}|$	4	$\left|T|+3|Z_{q}^{*}|+4|G\right|$	2
Ours	$2|Z_{q}^{*}|$	4	$\left|T|+|H|+2|Z_{q}^{*}|+3|G\right|$	1

**Table 6:** Security features.

Scheme	SF												
	1	2	3	4	5	6	7	8	9	10	11	12	13
[22]	√	×	√	√	×	√	√	×	×	×	√	×	×
[24]	√	√	√	√	√	√	×	×	×	×	√	×	√
[26]	√	√	√	√	√	√	√	√	√	√	√	×	√
[37]	√	√	√	√	√	√	×	×	√	√	×	×	√
[38]	√	√	√	√	√	√	×	×	√	√	√	×	√
Ours	√	√	√	√	√	√	√	√	√	√	√	√	√

## Data Availability

Data are contained within the article.
